# Special Issue “Lignocellulosic Biomass II”

**DOI:** 10.3390/molecules28176230

**Published:** 2023-08-24

**Authors:** Alejandro Rodríguez, Eduardo Espinosa, Carlos Martín

**Affiliations:** 1 BioPrEn Group, Instituto Químico para la Energía y el Medioambiente (IQUEMA), Chemical Engineering Department, Universidad de Córdoba, 14014 Córdoba, Spain; a02esvie@uco.es; 2 Department of Biotechnology, Inland Norway University of Applied Sciences, N-2317 Hamar, Norway; carlos.medina@inn.no; 3 Department of Chemistry, Umeå University, SE-901 87 Umeå, Sweden

## 1. Introduction

As a result of human population growth, the availability of residual lignocellulosic materials from agriculture, forestry, food- and wood-processing industries, and other waste streams is continuously increasing. The composition and abundance of lignocellulosic biomass make it a realistic option as feedstock for biorefineries, producing the bio-based fuels, materials, and chemicals required for the sustainable development of society without depending on fossil-based resources [1]. Lignocellulosic biomass represents a practically infinite feedstock source for satisfying the demands of the industry, and could thus contribute to achieving a carbon-neutral future and alleviating the current threats related to climate change. In previous collections, we compiled relevant contributions on the chemistry and technology of lignocellulosic biomass [2] and on the bioconversion of crop residues to biofuels and other bio-based products [3]. With this Special Issue, we present a new collection of articles dealing with cutting-edge topics in the field of lignocellulosic biomass.

## 2. This Issue

In this Special Issue, twelve original research papers and three reviews, covering some of the latest advances in research on lignocellulosic materials, are presented. Included in the collection are research results related to the by-products from agriculture and crop processing, e.g., corn stover, barley straw, rice husks, agave bagasse, apricot seed husks, walnut shells, cacao pods waste, and spent coffee grounds. The collection also features research on woody biomass, e.g., softwood and hardwood chips of different origin, teak wood, *Paulownia* bark, eucalyptus kraft pulp, and hardwood-based spent mushroom substrate, as well as herbaceous biomass, e.g., hemp and tall fescue.

Three articles deal with novel lignocellulosic materials of interest in biorefining. Wawro et al. [4] investigated the suitability of four Polish varieties of industrial hemp as raw materials for the production of lignocellulosic ethanol. After the alkaline treatment of samples of the four materials, biomass from the Tygra and Rajan varieties was submitted to enzymatic saccharification following either separated hydrolysis and fermentation or simultaneous saccharification and fermentation schemes. The authors concluded that both Tygra and Rajan varieties are promising raw materials for bioethanol production. Halysh et al. [5] evaluated apricot seed husks and walnut shells as potential raw materials for biorefineries in Ukraine. Both materials were pretreated with either H_2_SO_4_, NaOH, or by steam explosion, and then subjected to enzymatic saccharification with commercial cellulases. The results showed the potential of apricot seed husks and walnut shells for producing value-added products using affordable and environmentally friendly chemical technologies. It was concluded that alkaline pretreatment is the most advantageous method not only because of its glucose yield, but also because it allows an easy lignin regeneration from the spent liquor and the simple recovery of sodium hydroxide, which can be returned to the technological process. Batog et al. [6] investigated the use of annually renewable plant biomass from saline soils, namely tall fescue and hemp, for biorefining processes leading to biofuels and novel materials. After alkaline pretreatment and simultaneous saccharification, both tall fescue and hemp resulted in yields of 14 g ethanol per 100 g of raw material. Both types of halophyte biomass, were shown to be suitable as fillers for green composites displaying good mechanical properties.

Two papers bring up important aspects of the biorefinery processing of lignocellulosic biomass. In one of these, Poveda-Giraldo et al. [7] used data from the literature and simulation tools for developing a multi-criterion weighting of the best-performing schemes for the isolation of the main lignocellulose constituents. It was concluded that dilute acid is the most effective method for cellulose isolation and hemicelluloses removal, while the kraft process is the best option for lignin removal and its future use in biorefineries. The results are of high value for pretreatment systematization in the design of biorefineries. In the second of these papers, Allegretti et al. [8] combined hydrothermal pretreatment with a treatment with deep eutectic solvents in an environmentally friendly process for separating the main components of rice husks. The produced lignin fractions were characterized and evaluated as water reducers in cement pastes. The proposed strategy is very promising for the valorization of husks of raw and parboiled rice from a circular economy perspective.

Two papers address the extraction of bioactive compounds from lignocellulosic biomass. Rodríguez-Seoane et al. [9] applied pressurized extraction for recovering phenolics from *Paulownia* bark. Subcritical water extraction (SWE) was carried out under a non-isothermal operation by heating up to temperatures ranging between 140 and 240 °C, while supercritical CO_2_ (scCO_2_) extraction was performed at different pressures, temperatures, and ethanol concentrations. SWE reached higher extraction yield and antiradical capacity than scCO_2_ extraction. Extraction yields up to 30%, with extracts containing up to 7% phenolics, were achieved with SWE under heating to 160 °C while, at 240 °C, the yield decreased to 20%, but the phenolic content increased to 21% and the antiradical activity was considerably higher. Gallic acid, vanillic acid, vanillin, and apigenin were the major phenolics found in the extracts. Klausen et al. [10] evaluated the extraction of bioactive compounds and cellulose saccharification as valorization strategies for spent mushroom substrate (SMS). Four extraction methods, namely Soxhlet, reflux, SWE, and ultrasound-assisted extraction, were used. SWE at 150 °C resulted in the best extraction parameters among all the tested methods. Vanillic and chlorogenic acids were the primary phenolic acids identified in the extracts, and the concentration of caffeic acid correlated well with the antioxidant activity. The enzymatic saccharification of cellulose was enhanced after the extraction of bioactive compounds.

Three articles discussed the use of wood-processing products and agroindustrial residues for developing novel materials. Valente et al. [11] developed green composites by reinforcing biopolymeric matrices of poly(lactic acid) and poly(hydroxybutyrate) with micronized bleached eucalyptus kraft pulp fibers. The produced materials displayed superior mechanical performance and lower water uptake compared with the composites with non-micronized pulp fibers. The results showed the potential of micronization as a simple and sustainable alternative for the manufacturing of entirely bio-based composites. Naydenova et al. [12] used hydrolysis lignin, the residue of the industrial saccharification of woody materials, for producing biochars by hydrothermal liquefaction at 500–700 °C. The biochar produced at 600 and 700 °C displayed properties typical for microporous adsorbents, suitable for selective adsorption purposes. The authors also proposed to use the produced biochars as catalysts. Lavado-Meza et al. [13] present a study on producing biosorbents for the removal of heavy metals from aqueous solutions. The biosorbents were produced through the alkaline modification of coffee and cocoa agroindustrial waste. The adsorbtion capacity and other characteristics of the biosorbents were thoroughly assessed. Their efficiency on Pb (II) removal was higher than that of comparable biosorbents reported in the literature.

Two papers on the bioconversion of lignocellulosic materials are included in this Special Issue. Sierra-Ibarra et al. [14] reported on the fermentation of the hydrolysates of five different lignocellulosic materials with the ethanologenic strain *Escherichia coli* MS04. The hydrolysates contained different glucose and xylose concentrations. It was found that deleting the *xyIR* regulator plays an important role in xylose consumption and that acetate has a positive effect on the co-consumption rates of glucose and xylose in hydrolysates. Madubuike and Ferry [15] reported on the bioprospecting of novel enzymes for designing cocktails to deconstruct lignocellulose. They characterized a novel acetyl xylan esterase from the gut microbiota of the common black slug. The enzyme showed high thermal stability and potential for hydrolysing acetylated xylan.

Three review papers on crucial topics related to lignocellulose processing complete this Special Issue. Broda et al. [16] summarize the state of the art in bioethanol production from lignocellulose. The paper highlights the most challenging steps of the process, presents recent advances in the area, and discusses future perspectives for second-generation biorefineries. In a paper on processing biomass for hydrogen fermentation, Zhila et al. [17] review aspects related to the efficiency of hydrogen production through the fermentation of lignocellulosic hydrolysates. The effect of process parameters, both prior to and after fermentation, are discussed. The paper examines the formation of inhibitory compounds as a result of lignocellulose degradation during pretreatment and the effects of those compounds on the microorganisms involved in dark fermentation and photo-fermentation and on the management of post-fermentative liquid streams. A review on the durability of cellulosic-fibers-reinforced geopolymer composites (CFGC) is presented by Liu and Lv [18]. The paper analyzes the recent literature on the influence of nanomaterials on the properties of geopolymer composites. The effect of the degradation of cellulosic fibers and other factors on CFGC durability is summarized.

## 3. Conclusions

The content of this Special Issue shows the relevance of lignocellulosic biomass as a sustainable feedstock niche for bio-based industries. The guest editors of this Special Issue acknowledge all the contributing authors. Their contributions show the recent advancements in the research area of lignocellulosic biomass and provide indications of the routes to follow towards a bio-based society.

The heterogeneity of lignocellulosic biomass sources remains challenging for science and innovation today. New contributions regarding novel methods and technologies that would allow the efficient processing of different lignocellulosic materials are expected to be explored in a near future.
